# Immunogenic and Antioxidant Effects of a Pathogen-Associated Prenyl Pyrophosphate in *Anopheles gambiae*

**DOI:** 10.1371/journal.pone.0073868

**Published:** 2013-08-13

**Authors:** Bo G. Lindberg, Eleanor A. Merritt, Melanie Rayl, Chenxiao Liu, Ingela Parmryd, Berit Olofsson, Ingrid Faye

**Affiliations:** 1 Department of Molecular Biosciences, the Wenner-Gren Institute, Stockholm University, Stockholm, Sweden; 2 Royal Society of Chemistry, Thomas Graham House, Science Park, Cambridge, United Kingdom; 3 Science for Life Laboratory, Department of Medical Cell Biology, Uppsala University, Uppsala, Sweden; 4 Arrhenius Laboratory, Department of Organic Chemistry, Stockholm University, Stockholm, Sweden; Ecole Normale Supérieur de Lyon, France

## Abstract

Despite efficient vector transmission, *Plasmodium* parasites suffer great bottlenecks during their developmental stages within *Anopheles* mosquitoes. The outcome depends on a complex three-way interaction between host, parasite and gut bacteria. Although considerable progress has been made recently in deciphering *Anopheles* effector responses, little is currently known regarding the underlying microbial immune elicitors. An interesting candidate in this sense is the pathogen-derived prenyl pyrophosphate and designated phosphoantigen (*E*)-4-hydroxy-3-methyl-but-2-enyl pyrophosphate (HMBPP), found in *Plasmodium* and most eubacteria but not in higher eukaryotes. HMBPP is the most potent stimulant known of human Vγ9Vδ2 T cells, a unique lymphocyte subset that expands during several infections including malaria. In this study, we show that Vγ9Vδ2 T cells proliferate when stimulated with supernatants from intraerythrocytic stages of *Plasmodium falciparum* cultures, suggesting that biologically relevant doses of phosphoantigens are excreted by the parasite. Next, we used *Anopheles gambiae* to investigate the immune- and redox- stimulating effects of HMBPP. We demonstrate a potent activation *in vitro* of all but one of the signaling pathways earlier implicated in the human Vγ9Vδ2 T cell response, as p38, JNK and PI3K/Akt but not ERK were activated in the *A. gambiae* 4a3B cell line. Additionally, both HMBPP and the downstream endogenous metabolite isopentenyl pyrophosphate displayed antioxidant effects by promoting cellular tolerance to hydrogen peroxide challenge. When provided in the mosquito blood meal, HMBPP induced temporal changes in the expression of several immune genes. In contrast to meso-diaminopimelic acid containing peptidoglycan, HMBPP induced expression of dual oxidase and nitric oxide synthase, two key determinants of *Plasmodium* infection. Furthermore, temporal fluctuations in midgut bacterial numbers were observed. The multifaceted effects observed in this study indicates that HMBPP is an important elicitor in common for both *Plasmodium* and gut bacteria in the mosquito.

## Introduction

Human malaria is caused by *Plasmodium* parasites transmitted *via* *Anopheles* mosquitoes and results in more than 600,000 deaths annually [1]. To enable transmission in the mosquito, parasites ingested along with the blood meal must quickly undertake a complex series of developmental and invasive events (reviewed in 2). Several of these stages are associated with severe parasite population bottlenecks, mainly due to host immune responses triggered by both the parasite [3-5] and the growing midgut bacterial flora [6,7]. In accordance with the central dogma of insect immunity [8], the *Anopheles* immune system is thought to be activated through innate recognition of microbe associated molecular patterns (MAMPs) [7,9-12]. The identification of novel MAMPs is of great general value as it allows the fundamental mechanisms behind immune recognition of disease-causing microbes to be studied and elucidated. Although progress has been made in mapping *Anopheles* effector responses during the last decade or so, knowledge of the underlying immunological trigger molecules is still lagging behind. So far, two *Plasmodium* -derived putative MAMPs, glycosylphosphatidylinositol (*Pf*GPI) and hemozoin, have been investigated in *Anopheles* mosquitoes due to their immunogenic properties in mammals (reviewed in 13). We have previously shown that provision of *Pf*GPI in the blood meal induces the expression of antimicrobial peptides in *A. gambiae* [12]. In addition, both elicitors activate Akt and ERK signaling in cell lines and expression of the antiplasmodial factor nitric oxide synthase (NOS) in the midgut of *Anopheles* mosquitoes [10,11].

Prenyl pyrophosphates are small isoprenoid compounds found in all living cells where they are used as building blocks of isoprenoid end-products like sterols, dolichols and polyprenols. These metabolites are also known as phosphoantigens since they are recognized by Vγ9Vδ2 (also called Vγ2Vδ2) T cells, a lymphocyte subset unique for primates (reviewed in 14). The recognition leads to rapid cell activation, proliferation and secretion of cytokines and cytotoxic factors. While higher eukaryotes use the mevalonate pathway for isoprenoid synthesis, most bacteria and parasitic apicomlexan protozoa including *Plasmodium* use the 2-C-methyl-D-erythritol 4-phosphate (MEP) pathway. Vγ9Vδ2 T cells respond to metabolites from both pathways although with a clear preference for the MEP pathway metabolite (*E*)-4-hydroxy-3-methyl-but-2-enyl pyrophosphate (HMBPP) [15]. In accordance with Janeway’s model, the pathway restriction of HMBPP allows discrimination from self by the host. The only unresolved criteria for classifying HMBPP as a true MAMP in primates is the proof of a germline-encoded receptor interaction rather than with the somatically rearranged γδ T cell receptor (TCR) [16,17]. The γδ TCR has, however, been proposed to act as a pattern recognition receptor [18] and prenyl pyrophosphate recognition by Vγ9Vδ2 T cells is mediated *via* germline-encoded regions of the γδ TCR [19].

In this study, we used *Anopheles gambiae*, the main vector for *Plasmodium falciparum* in Sub-Saharan Africa, to investigate the immune stimulating properties of HMBPP in an invertebrate system. We found that HMBPP activates cell signaling in a comparable manner to Vγ9Vδ2 T cells and also displays effects on immune gene expression, redox status and midgut bacterial growth. To our knowledge, HMBPP is the first putative MAMP in common for *Plasmodium* and most bacteria. The diverse effects observed in this study indicate that HMBPP is an important key to understand the impact of the midgut bacteria on parasite transmission.

## Materials and Methods

### Ethics statements

This study was conducted in accordance with the principles expressed in the Declaration of Helsinki and was approved by the Ethical Review Board in Stockholm under reference number 2007/823-31/2 (later updated to 2011/850-32). Healthy blood donors were recruited from the Medical School, Uppsala University and venous blood collected anonymously in the lab by qualified personnel. In accordance with the ethical approval, the volunteers obtained an information leaflet and provided oral informed consent for the collection of anonymized blood samples and subsequent analysis.

### Chemicals

HMBPP was prepared in two steps as depicted in Figure S1 [20]. The reaction of commercially available 2-methyl-2-vinyloxirane (Figure S1 A) with TiCl_4_ at -84 °C furnished (*E*)-4-chloro-2-methylbut-2-en-1-ol (Figure S1 B). Subsequent treatment of the allylic chloride (Figure S1 B) with tris(tetra-*n*-butylammonium) hydrogen phosphate followed by purification of the crude reaction mixture by ion exchange chromatography and trituration with acetone yielded HMBPP (Figure S1 C) as the tris(ammonium) salt (*E:Z* 14:1). Isopentenyl pyrophosphate (IPP) was purchased from Sigma-Aldrich (St Louis, MO). Peptidoglycan (PGN; meso-diaminopimelic acid (DAP)-type from *Bacillus subtilis*) was kindly donated by Håkan Steiner, Stockholm University, Sweden. IL-2 was kindly provided by Giampietro Corradin, the University of Lausanne, Switzerland. RPMI, penicillin, streptomycin and glutamine were from Hyclone (Thermo Scientific, South Logan, UT, USA).

### Parasites

The FCR3 strain of *P. falciparum* (intracellular, asexual stages) was cultured using human type 0^+^ erythrocytes at 5% hematocrit in RPMI 1640 medium supplemented with human AB serum as described previously [21]. Cultures were regularly synchronized with sorbitol and tested for mycoplasma using the MycoAlert™ Mycoplasma Detection Kit (Lonza, Verviers, Belgium). Cell-free supernatants were prepared from early trophozoites at 10% parasitemia by 1.5 min centrifugation at 2,500 RPM and subsequent sterile filtering.

### Expansion of Vγ9Vδ2 T cells

Peripheral blood mononuclear cells (PBMCs) were prepared from the blood using Histopaque^®^-1077 (Sigma). The cells were counted in a Countess Automated Cell Counter (Invitrogen, Carlsbad, CA, USA) and expanded at 10^6^ per ml in RPMI medium supplemented with 2 mM L-glutamine, 100 U·ml^-1^ penicillin, 100 µg ml^-1^ streptomycin and 5% human AB serum (BioWhittaker, Lonza) at 37^°^ C in a humidified incubator under 5% CO_2_. An additional 2 mM L-glutamine was freshly added to the medium before the start of an experiment. The medium was mixed with supernatants from parasite-infected erythrocytes, prepared as described above. 10 U ml^-1^ rhIL-2 was added on day three. On days five and seven, 10 U ml^-1^ rhIL-2 was added together with media. On day 11, cells were counted and viability assessed using Trypan blue staining.

### Flow cytometry and mAbs

FITC-conjugated Abs to TCR Vδ2 (B6) were from BioLegend (San Diego, CA, USA) and PE-conjugated Abs to CD3 (UCHT1) were from Beckman Coulter (Brea, CA, USA). Flow cytometry was performed using a four colour FACSCalibur (BD Biosciences, San Jose, CA, USA). Data analysis was performed using FlowJo (Tree Star Inc., Ashland, OR, USA). Gating was done on the lymphocyte population, which for calculations on expansion index was equated with the viable cells obtained from the cell counting.

### Cell culture

The larvae-derived and immortalized hemocyte-like *A. gambiae* 4a3B cell line [22], was cultured at 27 ^°^C in Schneider’s modified *Drosophila* *medium* with L-glutamine (Lonza) supplemented with 10% fetal bovine serum (Sigma-Aldrich) and 100 U ml^-1^ penicillin–streptomycin mixture (Lonza).

### Mosquito rearing and experimental membrane feeding

*A. gambiae* mosquitoes (Keele strain), kindly provided by Hillary Hurd [23], were maintained at 27 °C and 80% humidity at a 12:12 (light: dark) photoperiod and provided a 10% sucrose solution. For feeding experiments, groups of 100-150 females (4–5 days old) were simultaneously membrane-fed (Hemotek 5W1 Membrane Feeding System, Discovery Workshops) a fresh blood meal containing either HMBPP (10 µM), PGN (10 µg ml^-1^) or *Aedes* physiological saline (APS; 13 mM NaCl, 0.5 mM KCl, 0.1 mM CaCl_2_) as a control.

### RNA extraction and Quantitative RT-PCR

From each cohort treatment, 5-10 fully engorged female mosquitoes were removed at 1, 3, 6 and 24 h post ingestion (hpi), homogenized and stored in TRIzol (Invitrogen). Similarly, midguts from 5–10 females per cohort treatment were at the different time points dissected on ice in APS and immediately immersed in TRIzol and homogenized. All samples were stored at -80 °C until RNA isolation and cDNA synthesis were performed. Total RNA was extracted according to manufacturer’s instructions and potential genomic DNA carryover was removed using DNAse I (Fermentas, Vilnius, Lithuania). First strand cDNA synthesis was performed using Ready-To-Go RT-PCR beads (GE Healthcare, Little Chalfont, UK). Quantitative PCR reactions were carried out using EvaGreen® dye (Solis BioDyne, Tartu, Estonia) in a LightCycler 480 instrument (Roche Diagnostics GmbH, Penzberg, Germany) and gene expression levels were normalized against the ribosomal S7 gene (see Table S1 for the list of primers). Data were analysed using the comparative 2^(-ΔΔCt)^ method to determine fold changes relative to blood fed controls. Quantification of bacteria was performed as previously described by Barillas-Mury and collaborators [24]. Briefly, midgut cDNA or genomic DNA isolated from midguts using the Gram positive protocol of the DNeasy blood and tissue kit (Qiagen) and samples were amplified using universal eubacteria 16S rRNA primers (Table S1) [12,25,26]. Measurements of each condition were repeated in technical triplicates. The amplification efficiencies were similar between target and reference genes.

### Western blot analysis

Cell stimulations and protein extractions were based on a slightly modified assay earlier described by Luckhart and collaborators [27]. Briefly, 2 x 10^6^ cells in 2 mL medium were plated in 12-well tissue culture plates and allowed to settle overnight. Stimulations were carried out by replacing the cell media with HMBPP (1 nM, 100 nM or 10 µM) or fresh control-medium. Following 10 min incubation, cells were washed in ice-cold PBS and harvested in lysis buffer (10 mM Tris–HCl pH 7.4, 1 mM EDTA, 100 mM NaCl, 1 mM NaF, 1 mM EGTA, 2 mM Na _3_VO_4_, 20 mM Na _4_P_2_O_7_, 0.1% SDS, 1% Triton X-100, 0.5% sodium deoxycholate, 1 mM phenylmethylsulfonyl fluoride, 10% glycerol, 60 µg ml^-1^ aprotinin, 10 µg ml^-1^ leupeptin, 1 µg ml^-1^ pepstatin, and 1 µg ml^-1^ calyculin A) while kept on ice under gentle shaking for 15 min. Cell debris was removed at 14,000 x g for 10 min and proteins were denatured by adding sample buffer to the resulting supernatants and heating at 80 ^°^C for 6 min. Proteins (30 µg per lane, as determined by BCA assay) were separated on 10% Bis-Tris gels and transferred to nitrocellulose membranes (Amersham, UK) using the NuPAGE SDS-PAGE gel system (Invitrogen). Membranes were blocked for 1 h with either 5% (w/v) non-fat dry milk or bovine serum albumin (BSA) in Tris-buffered saline containing 0.1% Tween 20 (TBST) and incubated at 4 ^°^C overnight with primary antibodies for phospho-ERK (1:10,000; Sigma Aldrich), phospho-JNK (1:1,250; Invitrogen), phospho-p38 (1:1,000; Cayman Europe, Tallinn, Estonia) or phospho-FOXO (1:1,000; Cell Signaling, Danvers, MA, USA). Membranes were washed and incubated at 4 ^°^C overnight with either HRP-conjugated goat anti-rabbit (Fab’) 2 (Invitrogen) at 1:20,000 (phospho-p38 and phospho-JNK) or at 1:5,000 (phospho-FOXO) or HRP-conjugated rabbit-anti mouse IgG (Sigma Aldrich) at 1:20,000 (phospho-ERK). To control for equal loading, membranes were incubated in stripping buffer (50 mM glycine (pH 2.3), 1 mM EDTA) for 30 min, washed, blocked with 5% BSA in TBST and re-probed with rabbit anti-GAPDH (1:10,000; Abcam, Cambridge, UK) followed by goat anti-rabbit (Fab’) 2 (1:20,000). Blots were incubated with SuperSignal West Pico Chemiluminescent Substrate (Thermo Scientific) and proteins were detected using a LAS-1000 CCD camera (Fujifilm, Tokyo, Japan). Relative band intensities were quantified and normalized against GAPDH using Multi Gauge v 3.0 image analysis software (Fujifilm).

### Oxidative stress analysis

To monitor changes in cellular tolerance to induced oxidative stress, confluent cells were seeded in 96-well plates at 2.5 x 10^4^ cells in 200 µL medium per well and allowed to settle overnight. Prior to oxidative insult, cells were pretreated with HMBPP or IPP and loaded with the ROS-sensitive fluorophores 2',7'-dichlorfluorescein-diacetate (DCFDA; 10 µM, Cayman Europe) or CellROX™ Deep Red Reagent (5 µM, Invitrogen). Following 0.5 h incubation with either probe, the cells were washed three times in complete medium and exposed to H_2_O_2_ (1 mM) in culture medium for 2 h in darkness. Relative fluorescence was measured using a FLUOstar Omega plate reader (BMG LABTECH, Ortenburg, Germany) with excitation filters at 490 or 630 nm and emission filters at 520 or 650 nm for DCFDA and CellROX respectively. Treatments were carried out in triplicates including a control for cell autofluorescence that was subtracted from each cohort’s mean value.

### Statistical analysis

Differences in relative expression between control and treatment groups in qRT-PCR and qPCR experiments were analysed using the 2^(Δ-CT)^ method and student’s unpaired t-test (two-tailed, equal variance). The Δ-CT data from qRT-PCRs were log-transformed before analysis. Differences in relative band intensities from western blots were analysed using a student’s unpaired t-test (two-tailed). Changes in oxidative stress were determined using one-way ANOVA with Dunnet’s pairwise comparison test against the control H_2_O_2_ treatment.

## Results

### Supernatants from infected erythrocytes triggers expansion of Vγ9Vδ2 T cells

**Figure 1:**
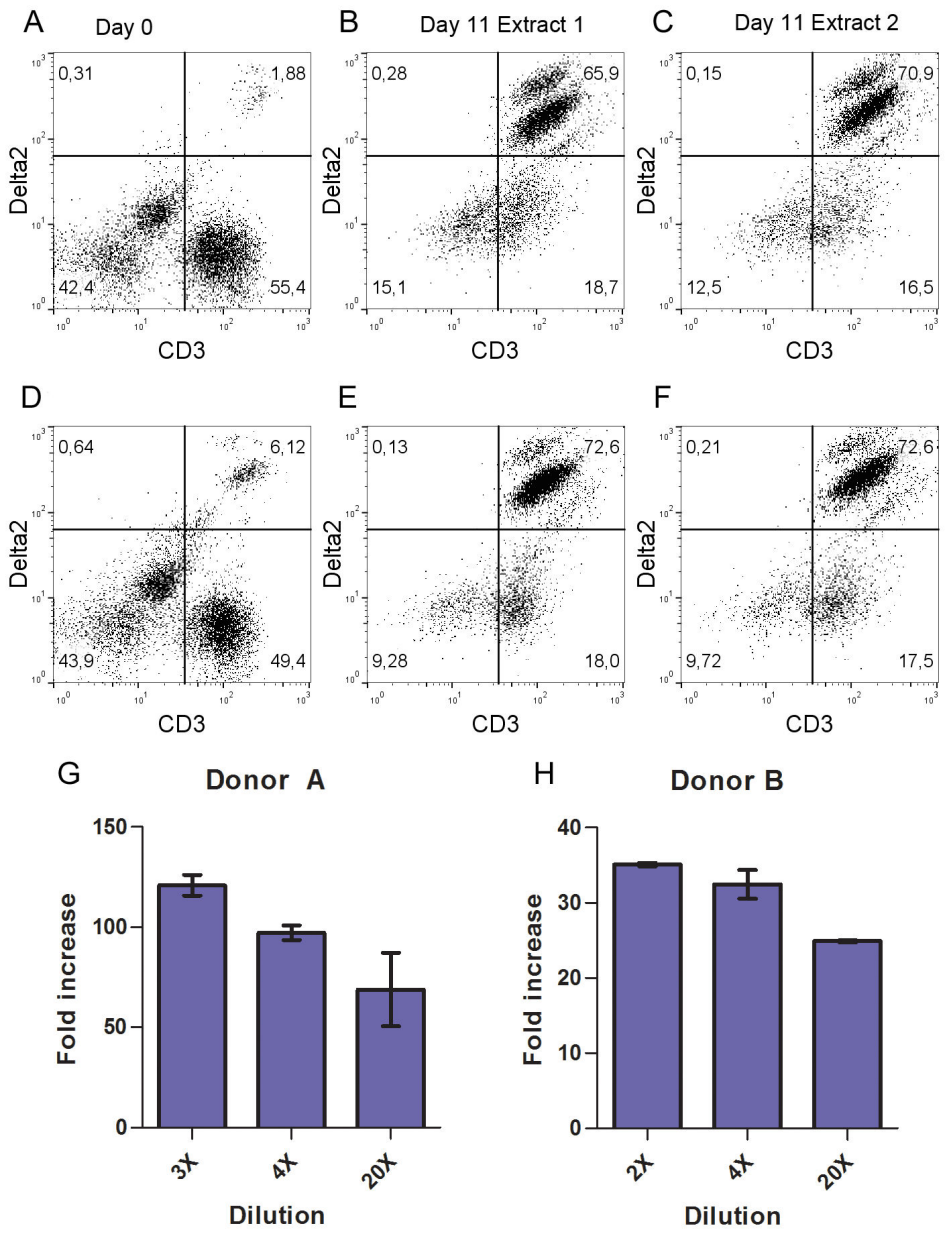
Vγ9Vδ2 T cells are greatly expanded by parasite-infected erythrocyte supernatants. PBMCs from two healthy donors were grown at 10^6^ cells per ml in the presence of diluted supernatants from parasite-infected erythrocytes. (**A**–**F**) Vγ9Vδ2 T cell expansion over time following stimulation with four times diluted supernatants (**G**–**H**) Fold increase of Vγ9Vδ2 T cells at 2, 3, 4 or 20 times dilutions of supernatants presented as means of duplicate samples ± SD.

Vγ9Vδ2 T cells are stimulated by phosphoantigens that are produced in *Plasmodium falciparum* [28]. To assess whether malaria-infected human blood is likely to contain parasite-derived phosphoantigens at quantities that may affect the mosquito, we isolated the supernatants from erythrocytes infected with *Plasmodium falciparum* parasites synchronized at the ring stage and incubated these with freshly isolated PBMCs. The fraction of Vγ9Vδ2 T cells of total T cells in PBMCs from donors A and B were 3.28% and 11.0% respectively (Figure 1 A, D). When the PBMCs were stimulated with four times diluted supernatants, the fraction of Vγ9Vδ2 T cells increased to around 80% for both donors with two different extracts (Figure 1 B-C, E-F). Dilution of the supernatants 2-20 X with fresh medium and incubation with PBMCs resulted in a thirty-fold and hundred-fold expansion of Vγ9Vδ2 T cells in eleven days for two different donors respectively (Figure 1 G-H). This is comparable to the expansion achieved with HMBPP as the stimulant (data not shown). In the absence of erythrocyte supernatants no Vγ9Vδ2 T cell expansion was observed. This strongly suggests that the concentration of phosphoantigen(s) excreted by parasite-infected erythrocytes is sufficient to induce a biological response.

### Effects on MAPK and PI3K signaling

**Figure 2:**
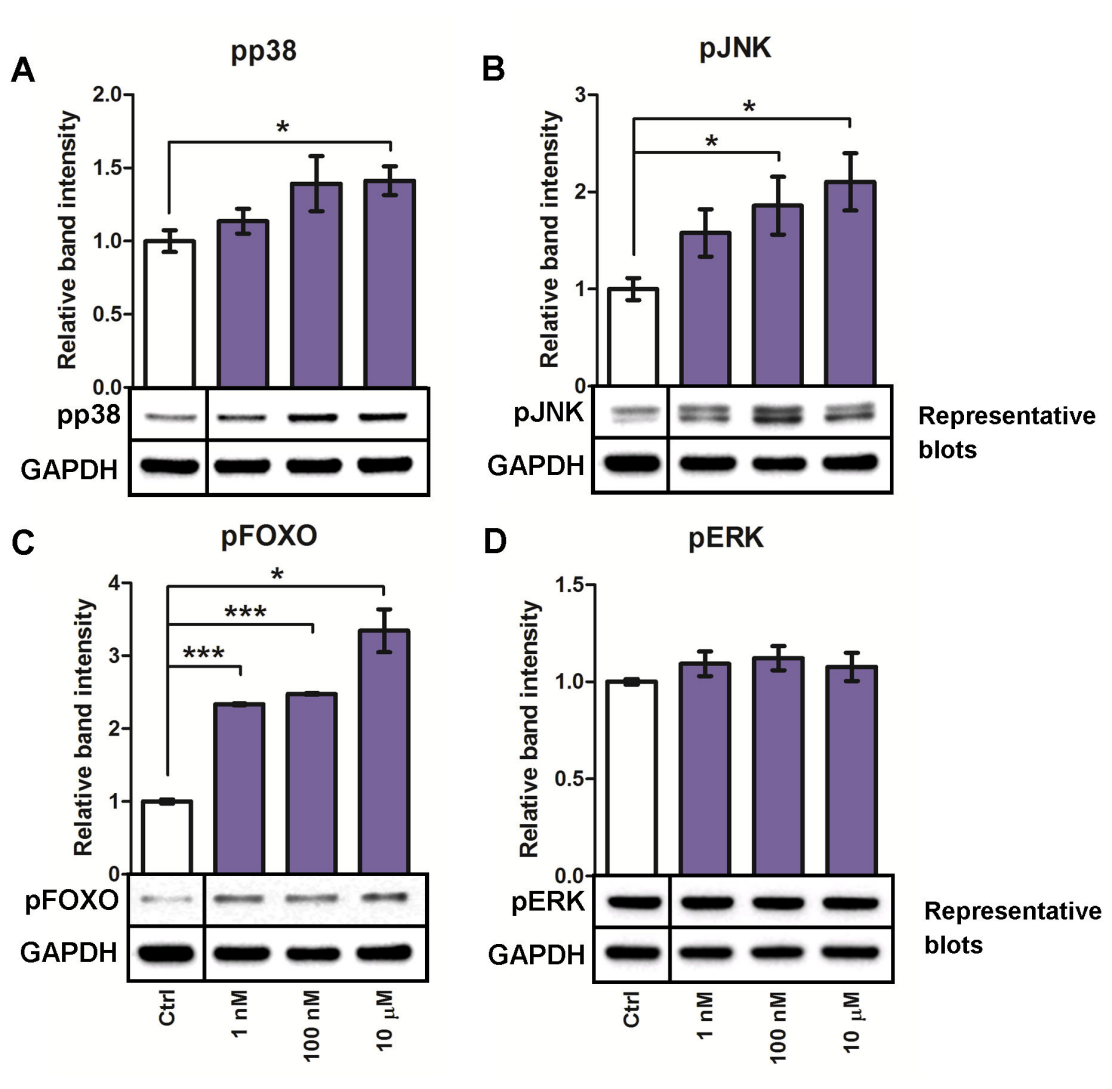
HMBPP activates phosphorylation of JNK, p38 and FOXO in *A. gambiae* 4a3B cells. (**A**–**D**) Mean fold changes ± SE relative to control stimulation and representative western blot images of target phosphoproteins. Membranes where stripped and reprobed for GAPDH to normalize relative band intensities. Asterisks represent significant differences (*P < 0.05, *** *P* < 0.001) from at least three independent experiments.

It has previously been shown that HMBPP stimulation rapidly induces TCR-associated responses through activation of JNK, p38 and the ERK, MAPKs, and PI3K signaling cascades in human Vγ9Vδ2 T cells [29]. Hence, we investigated whether homologous pathways in *A. gambiae* are activated in a similar fashion. The immune competent hemocyte-like 4a3B cell line was used to assay the response. Cells were stimulated with HMBPP at different concentrations for 10 min, a time point that overlaps the previously reported peak activation in Vγ9Vδ2 T cells (7-15 min). In a dose-dependent manner, HMBPP induced phosphorylation of JNK and p38 MAPKs as well as the transcription factor FOXO downstream of PI3K/Akt (Figure 2 A–C). Signaling through ERK was not affected by HMBPP (Figure 2 D). These findings indicate that mosquito cells are able to recognize and respond to HMBPP in a comparable, although not entirely equivalent manner as Vγ9Vδ2 T cells.

### Expression patterns of immune responsive genes

**Figure 3:**
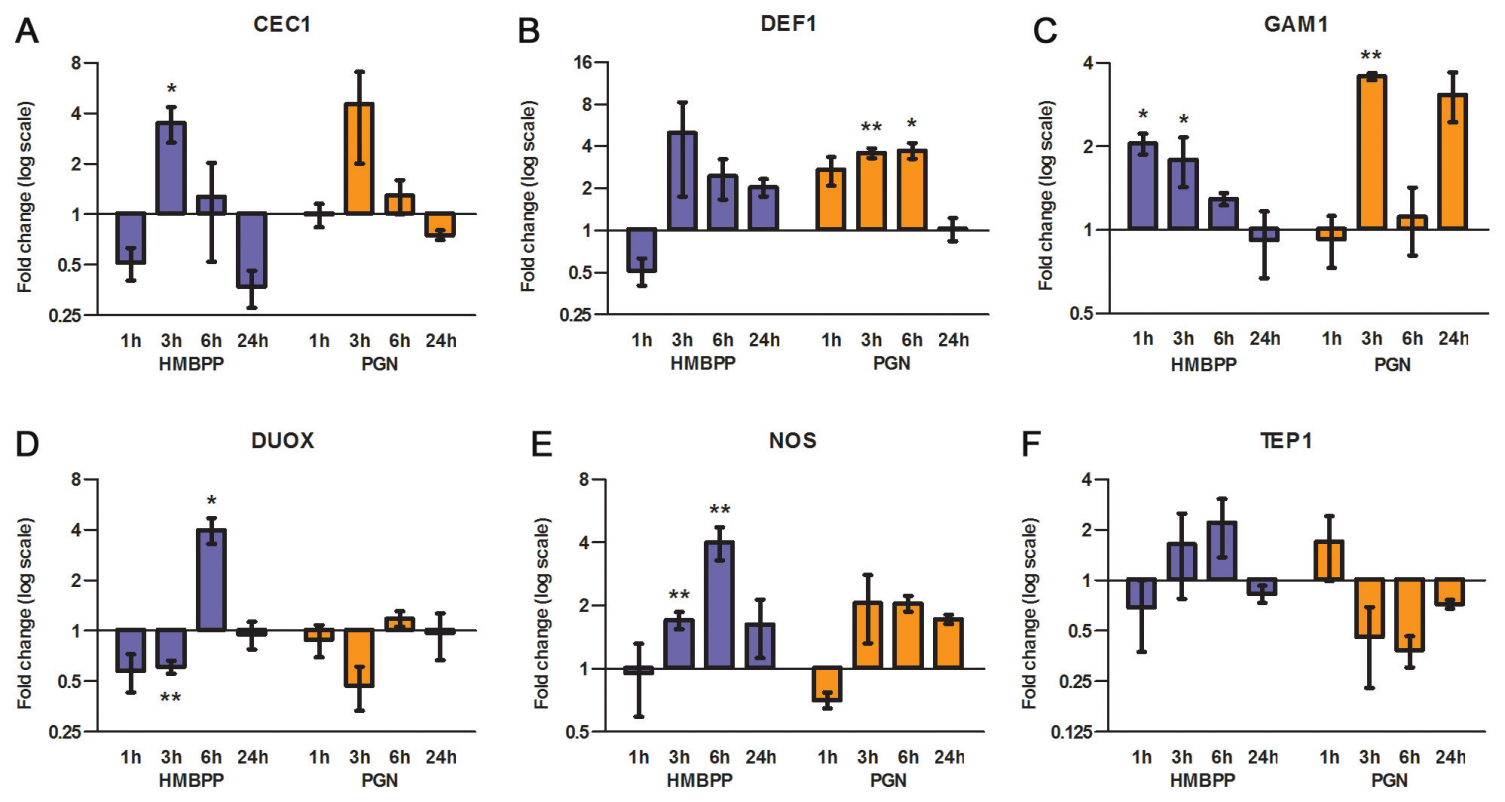
Temporal expression of selected immune genes relative to control blood meal as determined by qRT-PCR. (**A**–**E**) Bars represent mean ± SE from at least three (HMBPP) or two (PGN) independent experiments. Whole bodies were used from 5–10 fully engorged females. Asterisks (*P < 0.05, **P < 0.01) denote significantly different expression compared to control feed.

We next monitored the temporal expression profiles of six genes implicated in the *An. gambiae* immune response following addition of HMBPP in the blood meal. Before blood ingestion, arthropods inject their saliva which contains digestive enzymes. This includes apyrases that can dephosphorylate at least certain prenyl pyrophosphates [30]. Compared to the circulatory blood system in a human body, the small volume of blood in the artificial membrane feeder is prone to accumulation of components from mosquito saliva with successive feedings. To overcome this technical issue, we supplemented the blood with the highest dose of HMBPP used in the previous cell experiments (10 µM). A reference group was provided bacterial DAP-type PGN, the primary activator of humoral immune responses *via* the immunodeficiency (Imd) pathway in *Drosophila* and similarly thought to activate *A. gambiae* responses through interaction with peptidoglycan recognition protein LC (PGRPLC) [7]. Three AMPs, cecropin 1 (CEC1), defensin 1 (DEF1) and gambicin 1 (GAM1), two enzymes implicated in H_2_O_2_/NO-generation, dual oxidase (DUOX) and NOS, and thioester-containing protein 1 (TEP1) were assayed for transcription at 1, 3, 6 and 24 hpi. The overall response to HMBPP displayed both similarities to, and distinctions from the response to PGN (Figure 3). CEC1 expression was comparable with peak induction at 3 hpi followed by a quick decline to, or modestly below, control levels (Figure 3 A). DEF1 and GAM1 displayed different kinetics between the two treatments (Figure 3 B-C). Only bacterial PGN induced DEF1 significantly (3.6- and 3.7-fold at 3 and 6 hpi respectively), although HMBPP caused a stronger peak at 3 h (5-fold, *P* = 0.17). The mosquito-specific AMP, GAM1, was the only target gene upregulated at 1 hpi (2-fold) by HMBPP and declined over time unlike PGN treated mosquitoes where two peaks could be seen at 3 and 24 (not significant) hpi. Hence, both treatments upregulated AMP transcripts compared to control fed mosquitoes, although with different magnitudes and kinetics for two out of three targets. Only HMBPP induced DUOX and NOS transcription, both peaking (4-fold) at 6 hpi (Figure 3 E-F). In addition, a slight downregulation of DUOX was observed at 1 (not significant) and 3 hpi. No induction was observed of TEP1 by either treatment. Instead, a decrease in expression was observed in PGN treated mosquitoes, peaking at 6 hpi (2.7-fold).

**Figure 4:**
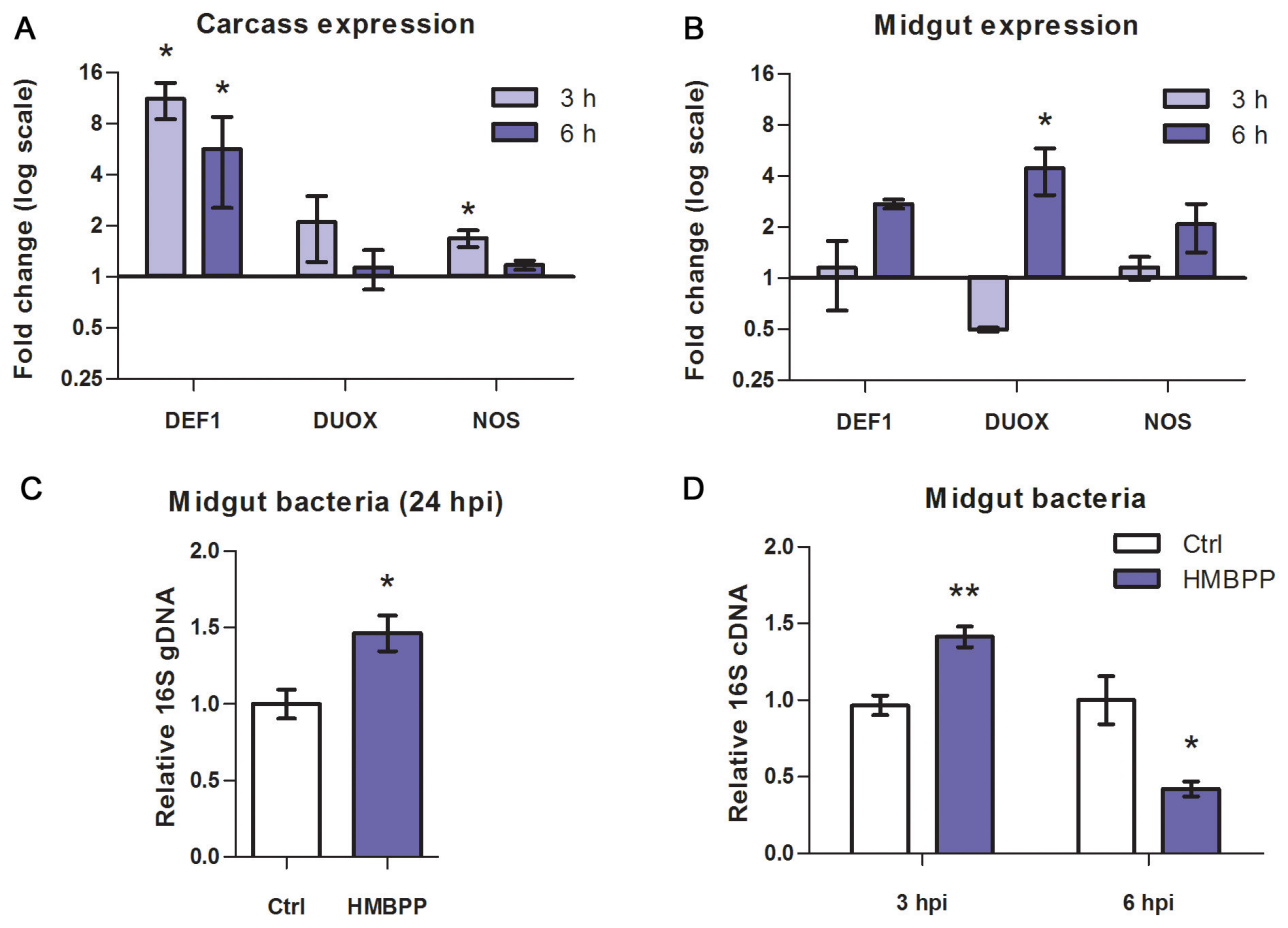
Expression in midgut and carcass and abundance of midgut bacteria relative to control feed. (**A**–**B**) At 3 and 6 hpi, 5-10 midguts and corresponding carcasses per sample were dissected and subjected to qRT-PCR analysis of DEF1, DUOX and NOS. (**C**–**D**) Effects of HMBPP on relative midgut bacterial 16S were quantified using qPCR. (**C**) At 24 hpi, bacterial gDNA was extracted from midguts (n = 4). (**D**) Midgut cDNA from 3 and 6 hpi (n = 3). Bars represent mean ± SE (*P < 0.05, ** *P* < 0.01).

As several of the selected genes were found to be upregulated by HMBPP, we investigated further whether this was due to a local epithelial response. Midgut and carcass samples were collected at 3 and 6 hpi and the expression of DUOX, NOS and DEF1 were quantified (Figure 4 A-B). In the midgut, all targets peaked at 6 hpi: modestly for NOS, 2.8-fold for DEF1 (*P* = 0.057) and significantly for DUOX (4.5-fold; Figure 4 A). Carcass expression of DUOX and NOS was not significantly altered by HMBPP treatment (Figure 4 B). DEF1, however, was strongly induced, peaking at 3 hpi (11.2-fold). Taken together, the data indicate that HMBPP induces immune gene expression in a unique manner both locally and systemically.

### Changes in midgut bacterial dynamics

As HMBPP induced mosquito immune genes we hypothesised that the blood meal supplementation would affect the quantity of the midgut bacterial flora. First, we performed qPCR with universal eubacterial 16S rRNA primers on midgut gDNA from mosquitoes at 24 hpi. This time point was selected as it crudely covers both the critical midgut penetration by *Plasmodium* ookinetes [31] and the midgut bacterial growth peak [26,32]. In mosquitoes fed with HMBPP, a 1.5-fold increase in bacteria was observed (Figure 4 C). To also monitor changes at earlier time points the assay was repeated using midgut cDNA from 3 and 6 hpi. Here, a 1.3-fold increase was observed followed by a 2.4-fold decline at 6 hpi (Figure 4 D). This suggests that HMBPP either directly or indirectly causes relative fluctuations of midgut bacterial numbers.

### HMBPP increases tolerance to H_2_O_2_-mediated oxidative stress *in vitro*

**Figure 5:**
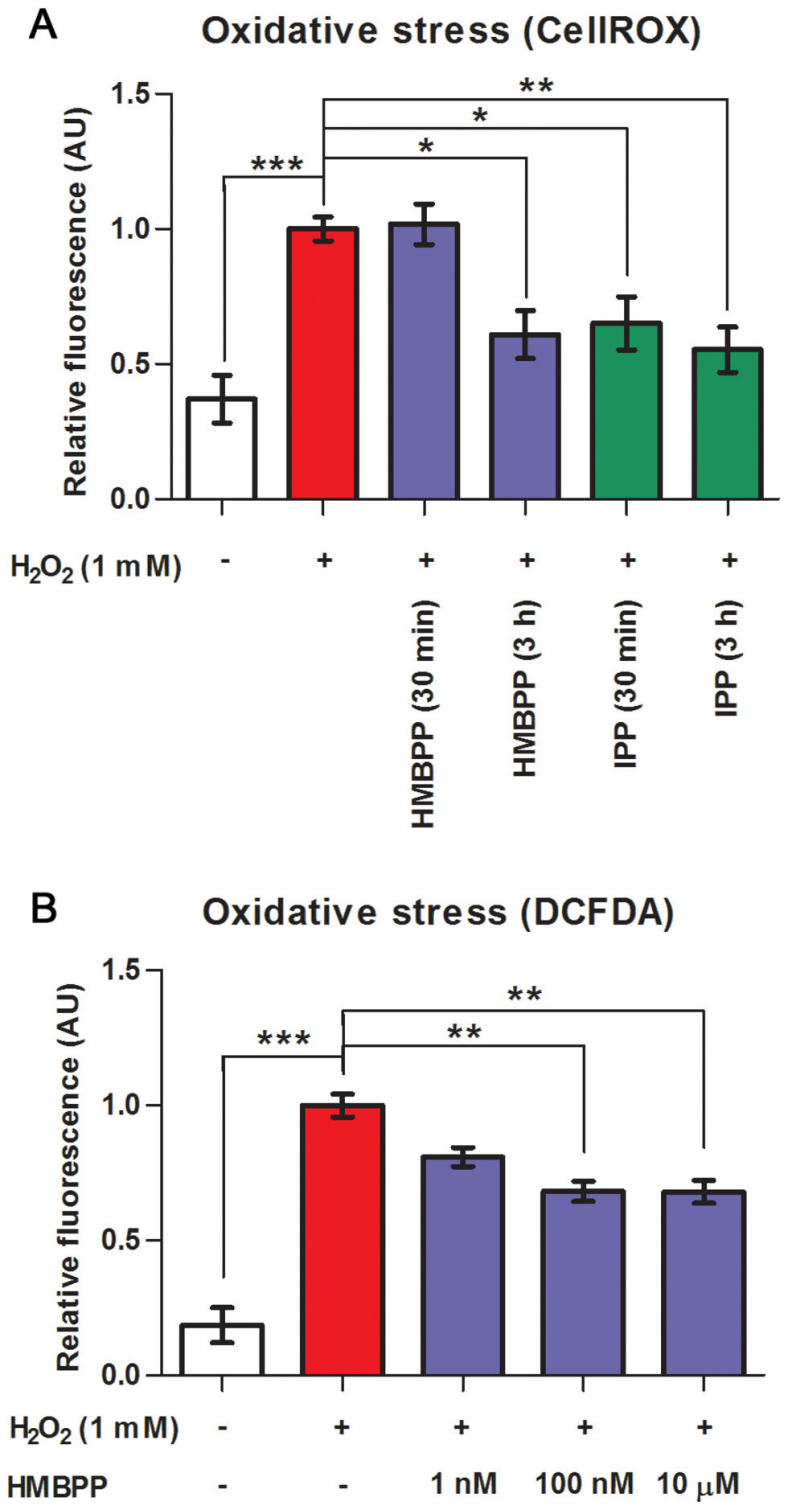
Prenyl pyrophosphate stimulation increased cellular tolerance to H_2_O_2_-induced oxidative stress. *A. gambiae* 4a3B cells were seeded in 96-well plates. (**A**) HMBPP or IPP (10 µM) were added at indicated time points prior to oxidative insult (n = 3). (**B**) Cells were preincubated for 3 h with HMBPP at different concentrations (n = 6-8). At 30 min before H_2_O_2_ addition, cells were loaded with (**A**) CellROX Deep Red Reagent or (**B**) DCFDA for oxidative stress detection. Cells were washed and challenged with H_2_O_2_ for 2 h. Bars represent mean relative fluorescence ± SE, asterisks denote significant differences (*P < 0.05, **P < 0.01, *** P < 0.001) compared to H_2_O_2_ control.

The induced changes in signal pathway activity and *in vivo* expression of proteins involved in ROS/RNS generation prompted us to investigate a putative relationship between these effects and changes in cellular redox state. Using a DCFDA-based *in vitro* assay with HMBPP at concentrations ranging from 1 nM to 10 µM we did not observe any changes over time compared to controls (data not shown). The universal isoprenoid precursor IPP downstream of HMBPP in the MEP pathway has earlier been reported to confer genoprotective effects against H_2_O_2_-mediated oxidative stress in human fibroblasts [33]. We therefore examined if IPP yielded similar effects in mosquito cells and whether HMBPP had comparable effects. Addition of IPP or HMBPP to the insect medium at 3 h prior to oxidative insult resulted in a 71% or 62% decrease in CellROX probe fluorescence relative to controls (Fig. 5 A). When added 30 min in advance only IPP showed an effect (a 55% decrease). Using 2’, 7’-dichlorofluorescein diacetate (DCFDA), a well-known reporter of general oxidative stress, a 3 h pretreatment of HMBPP resulted in a 39% decrease in probe fluorescence relative to controls (Figure 5 B). At 100 nM, but not 1 nM, an identical effect was observed suggesting a concentration in the nanomolar range for saturated bioactivity.

## Discussion

It is well known that *Plasmodium* parasites produce phosphoantigens. It has been established that Vγ9Vδ2 T cells can be stimulated by *Plasmodium* -infected erythrocytes [34] and by purified parasites from both schizont and merozoite stages of development [28,35]. Once the mosquito ingests infected blood, the bulk of parasites are, however, likely to be found within erythrocytes, making it relevant to test whether phosphoantigens are also released in sufficient quantities to instigate a biological response. As proof of concept, Vγ9Vδ2 T cells were stimulated with supernatants from parasite cultures synchronous for an intracellular stage. Our results demonstrate that the supernatants even at a 20X dilution contained sufficient phosphoantigen to stimulate the expansion of Vγ9Vδ2 T cells. Since HMBPP is approximately 10,000-fold more potent than any other phosphoantigen known [15], it is plausible that the observed effect was predominantly due to HMBPP released from infected erythrocytes. In further support of this, administered HMBPP yielded comparable expansion (Parmryd, unpublished data). To the best of our knowledge, no previous study has addressed whether phosphoantigens are excreted from parasite-infected erythrocytes.

The human Vγ9Vδ2 T cell response to HMBPP is mediated, at least in part, through immediate activation of JNK, ERK, p38 and PI3K/Akt signaling cascades [29]. In this study, we have shown that HMBPP stimulation of *A. gambiae* cells in a similar fashion induces rapid and potent phosphorylation of JNK, p38 MAPKs and also the transcription factor FOXO (Figure 2). Out of the MAPKs studied, ERK was the only protein with unaffected phosphorylation status even at the highest dose (10 µM). This indicates a distinct responsive pattern when compared to *Pf*GPI and hemozoin which both have been shown to induce ERK signaling in *Anopheles* [10,11]. Of note, the site-specific phosphorylation of FOXO (Thr24) promotes its cytoplasmic translocation and subsequent degradation. This indicates activation of PI3K/Akt signaling as phosphorylation at this site is carried out by activated Akt. Since insect cells lack TCRs which have previously been implicated in prenyl pyrophosphate recognition in Vγ9Vδ2 T cells [16], our findings indicate a possibly more ancient recognition of HMBPP mediated by innate, germline-encoded receptors in mosquitoes.

The oxidative status of *Anopheles* mosquitoes is an important determinant of *Plasmodium* susceptibility [36,37]. We found that supplementation of HMBPP in the blood meal of *A. gambiae* induces expression of both DUOX and NOS implicated in ROS/RNS generation (Figure 3). In *Drosophila* , DmDuox is directly involved in controlling proliferation of ingested bacteria in the gut through rapid generation of ROS [38]. Bacterial PGN was found to induce DmDuox expression but not activation. Increased ROS levels were, however, observed upon ingestion of PGN-depleted soluble bacterial extracts suggesting that activation of DmDuox is caused by one or several yet unidentified elicitors [39,40]. Our data indicate that HMBPP, but not PGN is a microbial trigger for DUOX in the midgut of *A. gambiae*. In support of this, HMBPP induced activation of p38 MAPK *in vitro* which mediates the microbe-triggered induction and activation of DmDuox in *Drosophila* [38].

In *A. gambiae*, DUOX has a paradoxical function, as it is required for the formation of a dityrosine matrix to decrease gut epithelial exposure to microbial immune elicitors after blood ingestion [24]. Knock down causes increased NOS expression and a dramatic parasite reduction. In line with this finding it is possible that also the midgut bacteria are regulated by DUOX and that the observed temporal changes in DUOX expression and midgut bacterial numbers are causally linked (Figures 3-4). Several studies have shown that the symbiotic bacteria harbouring the midgut affect *Plasmodium* infection rates, both indirectly [6,7] and more directly [41]. We found that HMBPP caused fluctuations in the numbers of midgut bacteria (Figure 4). At 24 hpi, a time point that roughly coincides with the growth peak of midgut bacteria [32], a 1.5-fold increase compared to control-fed mosquitoes was observed (Figure 4 C). During *Plasmodium* infection this event also overlaps with the ookinete midgut penetration, a stage that leads to the most severe parasite population bottleneck [42]. It is thus possible that the increased bacterial load observed at this critical time point affects the parasite.

MAPK and PI3K/Akt signaling have been associated with oxidative stress responses in a variety of species including *Anopheles* . Knock down experiments in adult female *A. gambiae* mosquitoes have indicated that JNK regulates the expression of H_2_O_2_ detoxifying enzymes and promotes survival during chronic oxidative stress [43]. It was recently demonstrated that H_2_O_2_ challenge causes phosphorylation of p38 in *A. gambiae* 4a3B cells [44] and FOXO (Akt- mediated) in *A* *. stephensi* ASE cells [45]. Inactivation of FOXO through the PI3K/Akt signal cascade could in turn also potentially increase oxidative stress [45]. It thus seemed plausible that the increased signal pathway activities as well as the in vivo effects on DUOX/NOS observed would correlate with changes in intracellular oxidative status. We investigated, but could not detect, any oxidative stress in 4a3B cells after exposure to HMBPP (data not shown). Instead, pretreatment with HMBPP increased tolerance to induced oxidative stress, albeit slower than IPP (Figure 5). Administered IPP has previously been suggested to protect against ROS through uptake and incorporation into the endogenous isoprenoid synthesis machinery of human fibroblasts [33]. The MEP pathway specificity and higher polarity of HMBPP, however, makes it unlikely to be utilized in isoprenoid synthesis or prenylation in higher eukaryotes. We speculate, hence, that the protective effect observed by HMBPP is indirect and requires induction, possibly *via* JNK signaling, of antioxidant genes.

Finally, our data suggest that HMBPP supplementation induce both local and systemic responses as in addition to DUOX in the midgut, DEF1 was induced in the carcass (Figure 3 A-B). In light of these findings, it is interesting that H_2_O_2_ and NO have earlier been suggested to mediate signaling between the midgut and abdominal tissues to promote systemic AMP expression during *Plasmodium* infection in *An. albimanus* [46]. In conclusion, this is to our knowledge the first study to demonstrate immunogenicity of a prenyl pyrophosphate in an invertebrate system.

## Supporting Information

Figure S1 **Two-step synthesis of HMBPP.**
(PDF)

Table S1 **List of probes used in this study.**
(XLS)

## References

[1] WHO (2012) World Malaria Report; WHO website available. http://www.who.int/malaria/publications/world_malaria_report_2012/en/. Accessed: 2013 January 14.

[2] Baton LA, Ranford-Cartwright LC (2005) Spreading the seeds of million-murdering death: metamorphoses of malaria in the mosquito. Trends Parasitol 21: 573-580. doi:10.1016/j.pt.2005.09.012. PubMed: 16236552. 16236552 10.1016/j.pt.2005.09.012

[3] Dimopoulos G, Seeley D, Wolf A, Kafatos FC (1998) Malaria infection of the mosquito *Anopheles gambiae* activates immune-responsive genes during critical transition stages of the parasite life cycle. EMBO J 17: 6115-6123. doi:10.1093/emboj/17.21.6115. PubMed: 9799221. 9799221 10.1093/emboj/17.21.6115 PMC1170938

[4] Dong YM, Aguilar R, Xi ZY, Warr E, Mongin E et al. (2006) *Anopheles gambiae* immune responses to human and rodent *Plasmodium* parasite species. PLOS Pathog 2: 513-525. PubMed: 16789837. 10.1371/journal.ppat.0020052 PMC1475661 16789837

[5] Vlachou D, Schlegelmilch T, Christophides GK, Kafatos FC (2005) Functional genomic analysis of midgut epithelial responses in *Anopheles* during *Plasmodium* invasion. Curr Biol 15: 1185–1195. doi:10.1016/j.cub.2005.06.044. PubMed: 16005290. 16005290 10.1016/j.cub.2005.06.044

[6] Dong YM, Manfredini F, Dimopoulos G (2009) Implication of the mosquito midgut microbiota in the defense against malaria parasites. PLOS Pathog 5: e1000423 PubMed: 19424427. 19424427 10.1371/journal.ppat.1000423 PMC2673032

[7] Meister S, Agianian B, Turlure F, Relógio A, Morlais I et al. (2009) *Anopheles gambiae* PGRPLC-mediated defense against bacteria modulates infections with malaria parasites. PLOS Pathog 5: e1000542 PubMed: 19662170. 19662170 10.1371/journal.ppat.1000542 PMC2715215

[8] Janeway CA (1989) Approaching the asymptote - evolution and revolution in immunology. Cold Spring Harb Symp Quant Biol 54: 1-13. doi:10.1101/SQB.1989.054.01.003. 10.1101/sqb.1989.054.01.003 2700931

[9] Dimopoulos G, Christophides GK, Meister S, Schultz J, White KP et al. (2002) Genome expression analysis of *Anopheles gambiae*: Responses to injury, bacterial challenge, and malaria infection. Proc Natl Acad Sci U S A 99: 8814-8819. doi:10.1073/pnas.092274999. PubMed: 12077297. 12077297 10.1073/pnas.092274999 PMC124381

[10] Lim JH, Gowda DC, Krishnegowda G, Luckhart S (2005) Induction of nitric oxide synthase in *Anopheles* stephensi by *Plasmodium falciparum*: Mechanism of signaling and the role of parasite glycosylphosphatidylinositols. Infect Immun 73: 2778-2789. doi:10.1128/IAI.73.5.2778-2789.2005. PubMed: 15845481. 15845481 10.1128/IAI.73.5.2778-2789.2005 PMC1087374

[11] Akman-Anderson L, Olivier M, Luckhart S (2007) Induction of nitric oxide synthase and activation of signaling proteins in *Anopheles* mosquitoes by the malaria pigment, hemozoin. Infect Immun 75: 4012-4019. doi:10.1128/IAI.00645-07. PubMed: 17526741. 17526741 10.1128/IAI.00645-07 PMC1952000

[12] Arrighi RBG, Debierre-Grockiego F, Schwarz RT, Faye I (2009) The immunogenic properties of protozoan glycosylphosphatidylinositols in the mosquito *Anopheles gambiae* . Dev Comp Immunol 33: 216-223. doi:10.1016/j.dci.2008.08.009. PubMed: 18822312. 18822312 10.1016/j.dci.2008.08.009

[13] Kumar S, Christophides GK, Cantera R, Charles B, Han YS et al. (2003) The role of reactive oxygen species on *Plasmodium* melanotic encapsulation in *Anopheles gambiae* . Proc Natl Acad Sci U S A 100: 14139-14144.Riley: Education Minnesota , Wahl S, Perkins DJ, Schofield L (2006) Regulating immunity to malaria. Parasite Immunol 28: 35-49 doi:10.1073/pnas.2036262100. PubMed: 14623973. 14623973 10.1073/pnas.2036262100 PMC283559

[14] Morita CT, Jin CG, Sarikonda G, Wang H (2007) Nonpeptide antigens, presentation mechanisms, and immunological memory of human Vgamma2Vdelta2 T cells: discriminating friend from foe through the recognition of prenyl pyrophosphate antigens. Immunol Rev 215: 59-76. doi:10.1111/j.1600-065X.2006.00479.x. PubMed: 17291279. 17291279 10.1111/j.1600-065X.2006.00479.x

[15] Puan KJ, Jin C, Wang H, Sarikonda G, Raker AM et al. (2007) Preferential recognition of a microbial metabolite by human Vgamma2Vdelta2 T cells. Int Immunol 19: 657-673. doi:10.1093/intimm/dxm031. PubMed: 17446209. 17446209 10.1093/intimm/dxm031

[16] Bukowski JF, Morita CT, Tanaka Y, Bloom BR, Brenner MB et al. (1995) Vgamma 2Vdelta2 TCR-dependent recognition of non-peptide antigens and Daudi cells analyzed by TCR gene transfer. J Immunol 154: 998-1006. PubMed: 7529807. 7529807

[17] Davey MS, Lin CY, Roberts GW, Heuston S, Brown AC et al. (2011) Human neutrophil clearance of bacterial pathogens triggers anti-microbial gamma delta T cell responses in early infection. PLOS Pathog 7: e1002040. 21589907 10.1371/journal.ppat.1002040 PMC3093373

[18] Morita CT, Mariuzza RA, Brenner MB (2000) Antigen recognition by human gamma delta T cells: pattern recognition by the adaptive immune system. Springer Semin Immunopathol 22: 191-217. doi:10.1007/s002810000042. PubMed: 11116953. 11116953 10.1007/s002810000042

[19] Wang H, Fang ZM, Morita CT (2010) Vgamma2Vdelta2 T cell receptor recognition of prenyl pyrophosphates is dependent on all CDRs. J Immunol 184: 6209-6222. doi:10.4049/jimmunol.1000231. PubMed: 20483784. 20483784 10.4049/jimmunol.1000231 PMC3069129

[20] Hecht S, Amslinger S, Jauch J, Kis K, Trentinaglia V et al. (2002) Studies on the non-mevalonate isoprenoid biosynthetic pathway. Simple methods for preparation of isotope-labeled (*E*)-1-hydroxy-2-methylbut-2-enyl 4-diphosphate. Tetrahedron Lett 43: 8929-8933. doi:10.1016/S0040-4039(02)02195-0.

[21] Trager W, Jensen JB (1976) Human malaria parasites in continuous culture. Science 193: 673-675. doi:10.1126/science.781840. PubMed: 781840. 781840 10.1126/science.781840

[22] Müller HM, Dimopoulos G, Blass C, Kafatos FC (1999) A hemocyte-like cell line established from the malaria vector *Anopheles gambiae* expresses six prophenoloxidase genes. J Biol Chem 274: 11727-11735. doi:10.1074/jbc.274.17.11727. PubMed: 10206988. 10206988 10.1074/jbc.274.17.11727

[23] Hurd H, Taylor PJ, Adams D, Underhill A, Eggleston P (2005) Evaluating the costs of mosquito resistance to malaria parasites. Evolution 59: 2560-2572. doi:10.1554/05-211.1. PubMed: 16526504. 16526504 PMC1602058

[24] Kumar S, Molina-Cruz A, Gupta L, Rodrigues J, Barillas-Mury C (2010) A peroxidase/dual oxidase system modulates midgut epithelial immunity in *Anopheles gambiae* . Science 327: 1644-1648. doi:10.1126/science.1184008. PubMed: 20223948. 20223948 10.1126/science.1184008 PMC3510679

[25] Kambris Z, Blagborough AM, Pinto SB, Blagrove MSC, Godfray HCJ et al. (2010) *Wolbachia* stimulates immune gene expression and inhibits *Plasmodium* development in *Anopheles gambiae* . PLOS Pathog 6: e1001143 PubMed: 20949079. 20949079 10.1371/journal.ppat.1001143 PMC2951381

[26] Nadkarni MA, Martin FE, Jacques NA, Hunter N (2002) Determination of bacterial load by real-time PCR using a broad-range (universal) probe and primers set. Microbiology 148: 257–266. PubMed: 11782518. 11782518 10.1099/00221287-148-1-257

[27] Surachetpong W, Singh N, Cheung KW, Luckhart S (2009) MAPK ERK signaling regulates the TGF-beta 1-dependent mosquito response to *Plasmodium falciparum* . PLOS Pathog 5: e1000366. 19343212 10.1371/journal.ppat.1000366 PMC2658807

[28] Behr C, Poupot R, Peyrat MA, Poquet Y, Constant P et al. (1996) Plasmodium falciparum stimuli for human gammadelta T cells are related to phosphorylated antigens of mycobacteria. Infect Immun 64: 2892-2896. PubMed: 8757809. 8757809 10.1128/iai.64.8.2892-2896.1996 PMC174163

[29] Correia DV, d’Orey F, Cardoso BA, Lança T, Grosso AR et al. (2009) Highly active microbial phosphoantigen induces rapid yet sustained MEK/Erk- and PI-3K/Akt-mediated signal transduction in anti-tumor human gamma delta T-cells. PLOS ONE 4: e5657. doi:10.1371/journal.pone.0005657. PubMed: 19479075. 19479075 10.1371/journal.pone.0005657 PMC2682580

[30] Vantourout P, Mookerjee-Basu J, Rolland C, Pont F, Martin H et al. (2009) Specific requirements for Vγ9Vδ2 T cell stimulation by a natural adenylated phosphoantigen. J Immunol 183: 3848-3857. doi:10.4049/jimmunol.0901085. PubMed: 19710470. 19710470 10.4049/jimmunol.0901085 PMC2809082

[31] Baton LA, Ranford-Cartwright LC (2004) *Plasmodium falciparum* ookinete invasion of the midgut epithelium of *Anopheles stephensi* is consistent with the Time Bomb model. Parasitology 129: 663–676. doi:10.1017/S0031182004005979. PubMed: 15648689. 15648689 10.1017/s0031182004005979

[32] Pumpuni CB, Demaio J, Kent M, Davis JR, Beier JC (1996) Bacterial population dynamics in three anopheline species: the impact on *Plasmodium* sporogonic development. Am J Trop Med Hyg 54: 214-218. PubMed: 8619451. 8619451 10.4269/ajtmh.1996.54.214

[33] Ling S, Wu YL, Zheng J, Linden J, Holoshitz J (2004) Genoprotective pathways - II. Attenuation of oxidative DNA damage by isopentenyl diphosphate. Mutat Resfund Mol M 554: 33-43. doi:10.1016/j.mrfmmm.2004.02.015. 10.1016/j.mrfmmm.2004.02.015 15450402

[34] Goodier M, Fey P, Eichmann K, Langhorne J (1992) Human peripheral blood gamma delta T cells respond to antigens of Plasmodium falciparum. Int Immunol 4: 33-41. doi:10.1093/intimm/4.1.33. PubMed: 1531764. 1531764 10.1093/intimm/4.1.33

[35] Goerlich R, Häcker G, Pfeffer K, Heeg K, Wagner H (1991) Plasmodium falciparum merozoites primarily stimulate the V gamma 9 subset of human gamma/delta T cells. Eur J Immunol 21: 2613-2616. doi:10.1002/eji.1830211045. PubMed: 1833205. 1833205 10.1002/eji.1830211045

[36] Kumar S, Christophides GK, Cantera R, Charles B, Han YS et al. (2003) The role of reactive oxygen species on *Plasmodium* melanotic encapsulation in *Anopheles gambiae* . Proc Natl Acad Sci U S A 100: 14139-14144. doi:10.1073/pnas.2036262100. PubMed: 14623973. 14623973 10.1073/pnas.2036262100 PMC283559

[37] Molina-Cruz A, Dejong RJ, Charles B, Gupta L, Kumar S et al. (2008) Reactive oxygen species modulate *Anopheles gambiae* immunity against bacteria and plasmodium. J Biol Chem 283: 3217-3223. PubMed: 18065421. 18065421 10.1074/jbc.M705873200

[38] Ha EM, Lee KA, Park SH, Kim SH, Nam HJ et al. (2009) Regulation of DUOX by the G alpha q-phospholipase C beta-Ca(2+) pathway in *Drosophila* gut immunity. Dev Cell 16: 386-397. doi:10.1016/j.devcel.2008.12.015. PubMed: 19289084. 19289084 10.1016/j.devcel.2008.12.015

[39] Ha EM, Lee KA, Seo YY, Kim SH, Lim JH et al. (2009) Coordination of multiple dual oxidase-regulatory pathways in responses to commensal and infectious microbes in *Drosophila* gut. Nat Immunol 10: 949-U919. doi:10.1038/ni.1765. PubMed: 19668222. 19668222 10.1038/ni.1765

[40] Ha EM, Oh CT, Bae YS, Lee WJ (2005) A direct role for dual oxidase in *Drosophila* gut immunity. Science 310: 847-850. doi:10.1126/science.1117311. PubMed: 16272120. 16272120 10.1126/science.1117311

[41] Cirimotich CM, Dong YM, Clayton AM, Sandiford SL, Souza-Neto JA et al. (2011) Natural microbe-mediated refractoriness to *Plasmodium* infection in *Anopheles gambiae* . Science 332: 855-858. doi:10.1126/science.1201618. PubMed: 21566196. 21566196 10.1126/science.1201618 PMC4154605

[42] Han YS, Thompson J, Kafatos FC, Barillas-Mury C (2000) Molecular interactions between *Anopheles stephensi* midgut cells and *Plasmodium berghei*: the time bomb theory of ookinete invasion of mosquitoes. EMBO J 19: 6030-6040. doi:10.1093/emboj/19.22.6030. PubMed: 11080150. 11080150 10.1093/emboj/19.22.6030 PMC305834

[43] Jaramillo-Gutierrez G, Molina-Cruz A, Kumar S, Barillas-Mury C (2010) The Anopheles gambiae oxidation resistance 1 (OXR1) gene regulates expression of enzymes that detoxify reactive oxygen species. PLOS ONE 17: e11168 PubMed: 20567517. 10.1371/journal.pone.0011168 PMC2887368 20567517

[44] Horton AA, Wang B, Camp L, Price MS, Arshi A et al. (2011) The mitogen-activated protein kinome from *Anopheles gambiae*: identification, phylogeny and functional characterization of the ERK, JNK and p38 MAP kinases. BMC Genomics 12: 574. doi:10.1186/1471-2164-12-574. PubMed: 22111877. 22111877 10.1186/1471-2164-12-574 PMC3233564

[45] Surachetpong W, Pakpour N, Cheung KW, Luckhart S (2011) Reactive oxygen species-dependent cell signaling regulates the mosquito immune response to *Plasmodium falciparum* . Antioxid Redox Sign 14: 943-955. doi:10.1089/ars.2010.3401. PubMed: 21126166. 10.1089/ars.2010.3401 PMC3042311 21126166

[46] Herrera-Ortiz A, Martínez-Barnetche J, Smit N, Rodriguez MH, Lanz-Mendoza H (2011) The effect of nitric oxide and hydrogen peroxide in the activation of the systemic immune response of *Anopheles* albimanus infected with Plasmodium berghei. Dev Comp Immunol 35: 44-50. doi:10.1016/j.dci.2010.08.004. PubMed: 20708028. 20708028 10.1016/j.dci.2010.08.004

