# Supplementary figures and images for: Immunogenic and Antioxidant Effects of a Pathogen-Associated Prenyl Pyrophosphate in Anopheles gambiae
Source: PLoS One. 2013 Aug 13;8(8):e73868. doi: 10.1371/journal.pone.0073868 (PMC3742518; doi:10.1371/journal.pone.0073868)

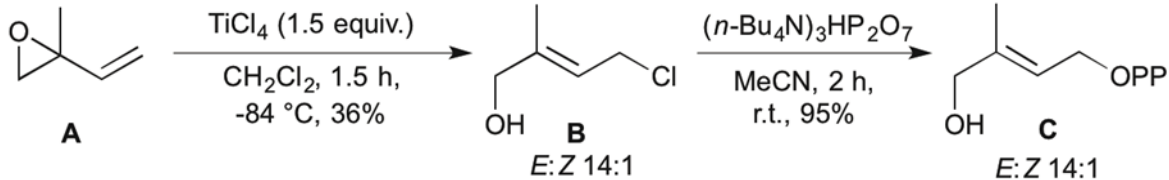

Supplement: Figure S1 — Two-step synthesis of HMBPP. (PDF) [file pone.0073868.s001.pdf]
